# The role of adiponectin in the association between abdominal obesity and type 2 diabetes: a mediation analysis among 232,438 Chinese participants

**DOI:** 10.3389/fendo.2024.1327716

**Published:** 2024-02-22

**Authors:** Lingjie He, Wenting Xuan, Dixing Liu, Jiana Zhong, Huijin Luo, Han Cui, Xiuwei Zhang, Weikun Chen

**Affiliations:** ^1^ Department of Endocrinology, The Tenth Affiliated Hospital, Southern Medical University (Dongguan People’s Hospital), Dongguan, China; ^2^ Department of Endocrinology, Dongguan Affiliated Hospital of Jinan University, Binhaiwan Central Hospital of Dongguan, Dongguan, China

**Keywords:** adiponectin, adiposity, abdominal obesity, type 2 diabetes mellitus, mediation analysis

## Abstract

**Background:**

Adiposity and adipokines are closely associated with obesity-related metabolic abnormalities, but little is known regarding whether abdominal obesity is linked to type 2 diabetes mellitus (T2DM) through circulating adiponectin levels. Thus, this large-population–based study was designed to investigate the mediating effect of adiponectin in the relationship between abdominal obesity and T2DM.

**Methods:**

A total of 232,438 adults who lived in Dongguan, Guangdong Province, China, were enrolled in the present study. The circulating adiponectin concentrations were measured using latex-enhanced immunoturbidimetric assay. The association between circulating adiponectin and other clinical parameters was detected by Spearman’s correlation analysis. Restricted cubic spline (RCS) regression was also used to address the non-linearity of the relationship between waist circumference and diabetes. Mediation analyses of circulating adiponectin were conducted using linear and logistic regression.

**Results:**

Subjects with abdominal obesity had lower levels of circulating adiponectin (*P* < 0.001). The circulating adiponectin value was inversely related to BMI (r = −0.370, *P* < 0.001), waist circumference (r = −0.361, *P* < 0.001), and fasting plasma glucose (r = −0.221, *P* < 0.001). The RCS plot showed a non-linear relation linking waist circumference with T2DM (*P* for non-linearity < 0.001). Patients with abdominal obesity presented 2.062 times higher odds of T2DM in comparison with those with non-abdominal obesity (odds ratio, 2.062; 95% confidence interval, 1.969–2.161) after adjusting for confounders. In the mediation analyses, the circulating adiponectin mediated the association between abdominal obesity and T2DM, with a mediation effect of 41.02% after adjustments. The above results were consistent in both men and women.

**Conclusion:**

The relationship between abdominal obesity and T2DM is mediated through circulating adiponectin level in adults, suggesting that circulating adiponectin might be a potential predictor for controlling the adverse progression from adiposity to T2DM.

## Introduction

Type 2 diabetes mellitus (T2DM) is becoming a serious public health crisis affecting 783.2 million adults worldwide by 2045 and imposing a substantial burden on the healthcare system (1). In recent decades, lifestyle modification and, consequently, excess body fat accumulation have triggered obesity epidemic, which, in turn, have contributed to the increase in diabetes and diabetes-related conditions (2). Thus, obesity is summarized as a leading risk factor for T2DM (3, 4). Despite the clear and significant obesity–T2DM association, the exact mechanism that connects these conditions has not been fully elucidated. In recent years, basic scientific studies have attempted to explore the pathogenesis involved in a variety of obesity-induced processes able to favor the development and progression of T2DM. Among them, the dysregulation of adipose tissue and the aberrant secretion of adipokine induced by obesity have been established as the forefront of the relation between obesity and diabetes, which emerged as one of the major underlying mechanisms of T2DM (5).

Adipose tissue has been documented as not only a primary site of energy storage but also an endocrine organ releasing a wide array of adipokines, which are closely linked to metabolic disease (6). Among them, adiponectin, the most abundant adipokine synthesized and secreted from fatty tissue, has gained much attention recently. Adiponectin has been recognized as a metabolically favorable adipokine, whose reduction plays a crucial role in both obesity and T2DM (7–9). Previous studies have shown that adiponectin has many beneficial metabolic effects, particularly lipid metabolism–regulating, insulin-sensitizing, and anti-inflammatory properties, which might be the potential roles that are linked to adiponectin in protecting from obesity-related diseases (10, 11). Ghoshal et al. showed that adiponectin played a crucial role in diabetic dyslipidemia by regulating glucose levels and fatty acid breakdown (12). Moreover, accumulative clinical evidence also indicated that the circulating adiponectin levels were negatively correlated with insulin resistance/T2DM and metabolic syndrome in human subjects (13–16).

As mentioned above, adiponectin secreted from adipose tissue has effects on obesity-related insulin resistance and T2DM. However, whether abdominal obesity is linked to T2DM through adiponectin remains unknown in humans. Thus, we sought to explore whether the association between abdominal obesity and T2DM is mediated by adiponectin and then further evaluate the degree of mediating effect in Chinese population.

## Methods

### Study population

**Figure 1:**
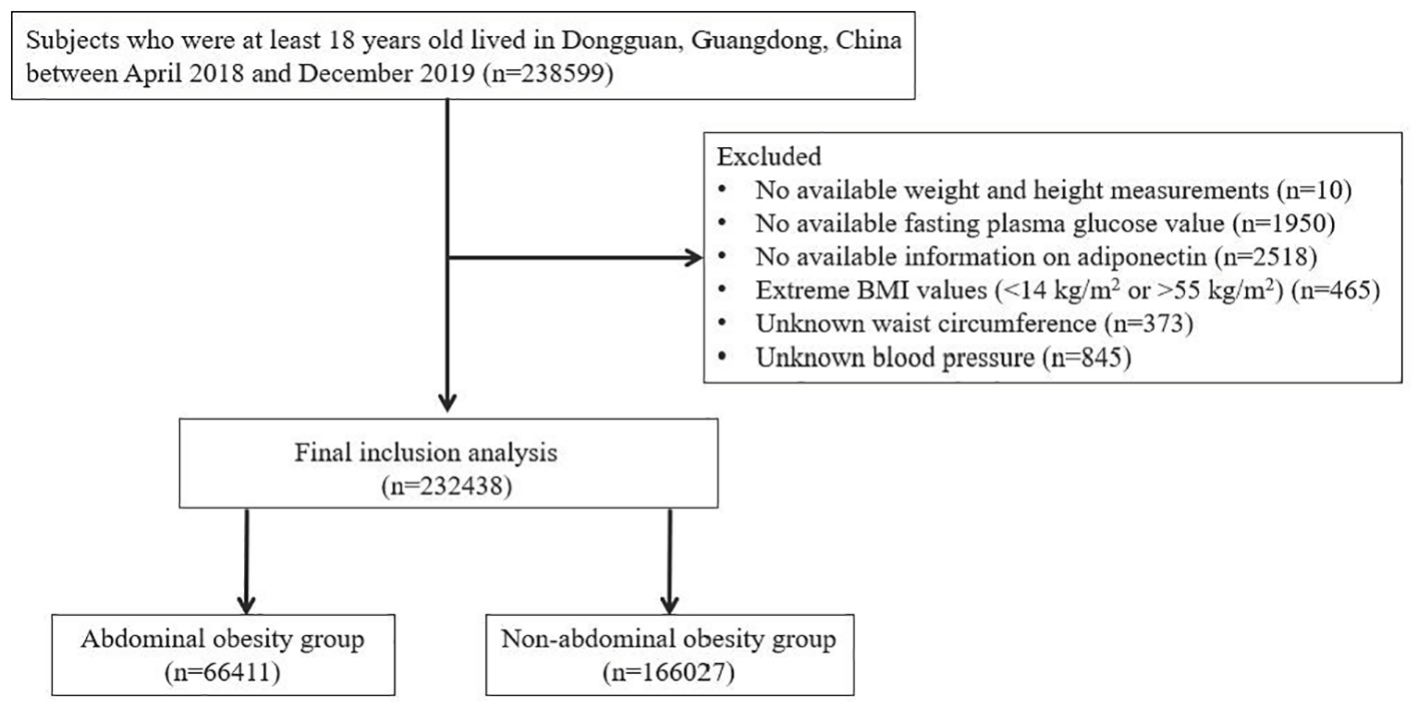
Flow chart of the study design.

This study recruited a representative sample of community dwelling members who lived in Dongguan, Guangdong Province, China, from 2021 to 2022. Analyses were based on the selected sample with available clinical data and diabetic status. From the initial sample of 238,965 Chinese population, we identified 238,599 participants aged 18 years and older. Those with missing information on adiponectin, fasting blood glucose, and blood pressure were excluded from further analysis. Moreover, we also excluded those without data on anthropometric measurements at baseline. Overall, 232,438 participants were included, and the sample selection for the analysis was described in **Figure 1**. This study obtained ethical approval from The Tenth Affiliated Hospital of Southern Medical University (Dongguan People’s Hospital). Each participant gave a written informed consent.

### Clinical and biochemical measurements

The standardized questionnaires were applied to obtain the age, gender, and medical history of all participants. Each questionnaire was filled out by personnel who have received uniformly trained. The anthropometric parameters, including waist circumference, weight, height, and blood pressure were used in the final analysis. Waist circumference of each participant was measured as the midpoint between the lower costal border and the roof of the iliac crest. Body mass index (BMI) was calculated as body weight (kg)/height (m^2^). Blood pressure was assessed after resting for at least 5 min using an electronic sphygmomanometer. Fasting plasma glucose (FPG) was determined using the glucose oxidase method after 12 h of fasting.

### Measurement of adiponectin

Circulating adiponectin concentrations were measured by a latex-enhanced immunoturbidimetric assay, following the manufacturer’s protocol. The kits were kindly provided by Guangdong Uniten Biotechnology Co., Ltd (NMPA registration number: 20182400947). The dynamic range of the adiponectin assay is from 2 to 40.0 µg/mL, with a detection sensitivity at 0.5 µg/mL and the inter/intra-assay coefficients of variation less than 5%.

### Assessment of type 2 diabetes mellitus

The diabetes mellitus was defined as FPG ≥7.0 mmol/L or a self-reported history of diabetes. Each study participant was invited to respond to the following question: “Have you ever been diagnosed with diabetes by a doctor?”, and patients with a positive response were considered as diabetic. Otherwise, the FPG ≥7.0 mmol/L also confirms diabetes. In these cases, the type of diabetes was not available, but, as type 1 diabetes is significantly less prevalent and most often occurs in young adults or children, we considered that the majority of incident cases in this study were T2DM.

### Statistical analyses

The characteristics of the participants were classified according to abdominal obesity status. Quantitative variables were presented as mean ± standard deviation (SD) or as median (Q1–Q3). In addition, the qualitative variables were expressed as frequencies (percentages). Statistical differences between the two groups were assessed by Student’s T-tests for normally distributed data and Mann–Whitney U-tests for skewed data. The chi-square tests were carried out for categorical variables. Then, Spearman’s correlation analysis was used to evaluate the correlations between adiponectin and other clinical variables. Moreover, logistic regression analyses were also performed to address the association between abdominal obesity and T2DM. The results were presented as odds ratio (OR) and 95% confidence interval (CI). Restricted cubic spline (RCS) regression was also used to evaluate any potential linearity or non-linearity of the relationship between waist circumference and diabetes.

In this study, general causal mediation analysis was constructed to distinguish the direct effect of abdominal obesity on the risk of T2DM and the indirect effect mediated by adiponectin. Total effect is the effect of abdominal obesity on T2DM without adiponectin. Direct effect is defined as the effect of abdominal obesity on T2DM when adiponectin is included in the model as a covariate. Indirect effect is the effect of abdominal obesity on T2DM via adiponectin. The proportion mediated (%) is the proportion of the effect of abdominal obesity on T2DM that goes via generalized adiponectin. Linear regression models and logistic regression were applied to calculate the standardized regression coefficient (β). The mediation analysis model was presented in **Supplementary Figure 1**. In the mediation effect model, the predictor variable (X) was abdominal obesity; the mediator variable (M) was adiponectin; the outcome variable (Y) was T2DM. β_Tot_ (total effect) is the coefficient relating X to Y. β_1_ (indirect effect 1) is the coefficient relating X to M. β_2_ (indirect effect 2) is the coefficient relating M to Y after adjusting for X. β_Dir_ (direct effect) is the coefficient relating X to Y adjusted for M. The total indirect effect was calculated as β_1_ × β_2_. The proportion of mediation effect (%) = total indirect effect/total effect × 100. In mediation analysis, testing the significance of the mediation effect is equivalent to testing the null hypothesis H_0_: β_1_ × β_2_ = 0 versus the alternative hypothesis H_a_: β_1_ × β_2_ ≠ 0, by using the Bootstrap tests. Statistical analysis was carried out using SPSS 26.0 (IBM Corporation, Armonk, NY, USA). *P-*value <0.05 was considered to be statistically significant.

## Results

### Population characteristics

This studied sample consisted of 232,438 participants (mean age, 42.6 years; 44.9% men). The average BMI (SD) was 23.9 (3.5) kg/m^2^, and the median of adiponectin was 4.9 µg/mL (IQR, 2.6). The prevalence of T2DM was 3.9% and that for hypertension was 9.8%. Among the included population, 66,411 (28.6%) have abdominal obesity and 166,027 (71.4%) have non-abdominal obesity. Overall, adults with abdominal obesity were older, were more often men, have a higher family history of diabetes, and were more likely to present a higher prevalence of comorbidities. Moreover, higher BMI, waist circumference, blood pressure, and FPG were more prevalent among adults with abdominal obesity (**Table 1**). However, the level of circulating adiponectin was significantly lower in patients with abdominal obesity than that in their counterparts. The median of adiponectin was 4.2 µg/mL for abdominal obesity subjects and 5.2 µg/mL for participants without abdominal obesity (*P* < 0.001).

**Table 1:** Characteristics of the study participants.

Variables	All subjects (n = 232,438)	Abdominal obesity (n = 66,411)	Non-abdominal obesity (n = 166,027)	*P*-value
Age (years)	42.6 ± 5.6	43.3 ± 5.6	42.4 ± 5.7	<0.001
Gender, n (%)				<0.001
Male	104,274 (44.9)	39,283 (59.2)	64,991 (39.1)	
Female	128,164 (55.1)	27,128 (40.8)	101,036 (60.9)	
Height (cm)	161.9 ± 7.8	164.3 ± 8.2	160.9 ± 7.5	<0.001
Weight (kg)	63.0 ± 11.8	73.8 ± 11.0	58.7 ± 9.0	<0.001
BMI (kg/m^2^)	23.9 ± 3.5	27.3 ± 3.2	22.6 ± 2.7	<0.001
Waist circumference (cm)	82.9 ± 10.3	94.7 ± 8.3	78.2 ± 6.5	<0.001
SBP (mmHg)	122.6 ± 14.5	127.9 ± 15.0	120.4 ± 13.7	<0.001
DBP (mmHg)	78.9 ± 10.3	82.6 ± 10.9	77.4 ± 9.6	<0.001
FPG (mmol/L)	5.1 (4.6–5.5)	5.2 (4.7–5.7)	5.0 (4.6–5.4)	<0.001
Adiponectin (µg/mL)	4.9 (3.8–6.4)	4.2 (3.3–5.3)	5.2 (4.0–6.7)	<0.001
T2DM, n (%)				<0.001
Yes	8,960 (3.9)	4,644 (7.0)	4,316 (2.6)	
No	223,478 (96.1)	61,767 (93.0)	161,711 (97.4)	
Hypertension, n (%)				<0.001
Yes	22,670 (9.8)	11,629 (17.5)	11,041 (6.7)	
No	209,361 (90.2)	54,685 (82.5)	154,676 (93.3)	
Coronary atherosclerotic heart disease, n (%)				<0.001
Yes	719 (0.3)	321 (0.5)	398 (0.2)	
No	231,264 (99.7)	65,959 (99.5)	165,305 (99.8)	
Stroke, n (%)				<0.001
Yes	421 (0.2)	160 (0.2)	261 (0.2)	
No	231,544 (99.8)	66,117 (99.8)	165,427 (99.8)	
Family history of diabetes, n (%)				<0.001
Yes	26,353 (12.1)	8,737 (14.0)	17,616 (11.3)	
No	191,933 (87.9)	53,647 (86.0)	138,286 (88.7)	

BMI, body mass index; SBP, systolic blood pressure; DBP, diastolic blood pressure; FPG, fasting plasma glucose.

Abdominal obesity was defined as waist circumference ≥85 cm in women and ≥90 cm in men.

### Relationship between circulating adiponectin and other variables

The association between adiponectin and clinical parameters in all participants and by gender was shown in **Table 2**. Of the whole study population, Spearman’s correlation analysis revealed that the adiponectin value was significantly related to BMI (r = −0.370, *P* < 0.001) and waist circumference (r = −0.361, *P* < 0.001). Moreover, FPG, diastolic blood pressure (DBP), and systolic blood pressure (SBP) were also inversely correlated with adiponectin (all *P* < 0.001), whereas age exhibited a weak positive association with adiponectin (r = 0.072, *P* < 0.001). The results of Spearman’s correlation analysis between adiponectin and other clinical parameters were consistent in both men and women.

**Table 2:** Spearman’s correlation analysis of adiponectin and other variables.

Variables	All subjects		Male		Female	
	r	*P*-value	r	*P-*value	r	*P*-value
Age	0.072	<0.001	0.088	<0.001	0.075	<0.001
BMI	−0.370	<0.001	−0.352	<0.001	−0.298	<0.001
Waist circumference	−0.361	<0.001	−0.308	<0.001	−0.257	<0.001
Systolic blood pressure	−0.163	<0.001	−0.126	<0.001	−0.089	<0.001
Diastolic blood pressure	−0.167	<0.001	−0.134	<0.001	−0.088	<0.001
FPG	−0.221	<0.001	−0.224	<0.001	−0.206	<0.001

BMI, body mass index; FPG, fasting plasma glucose.

### Relationship between abdominal obesity and T2DM

**Figure 2:**
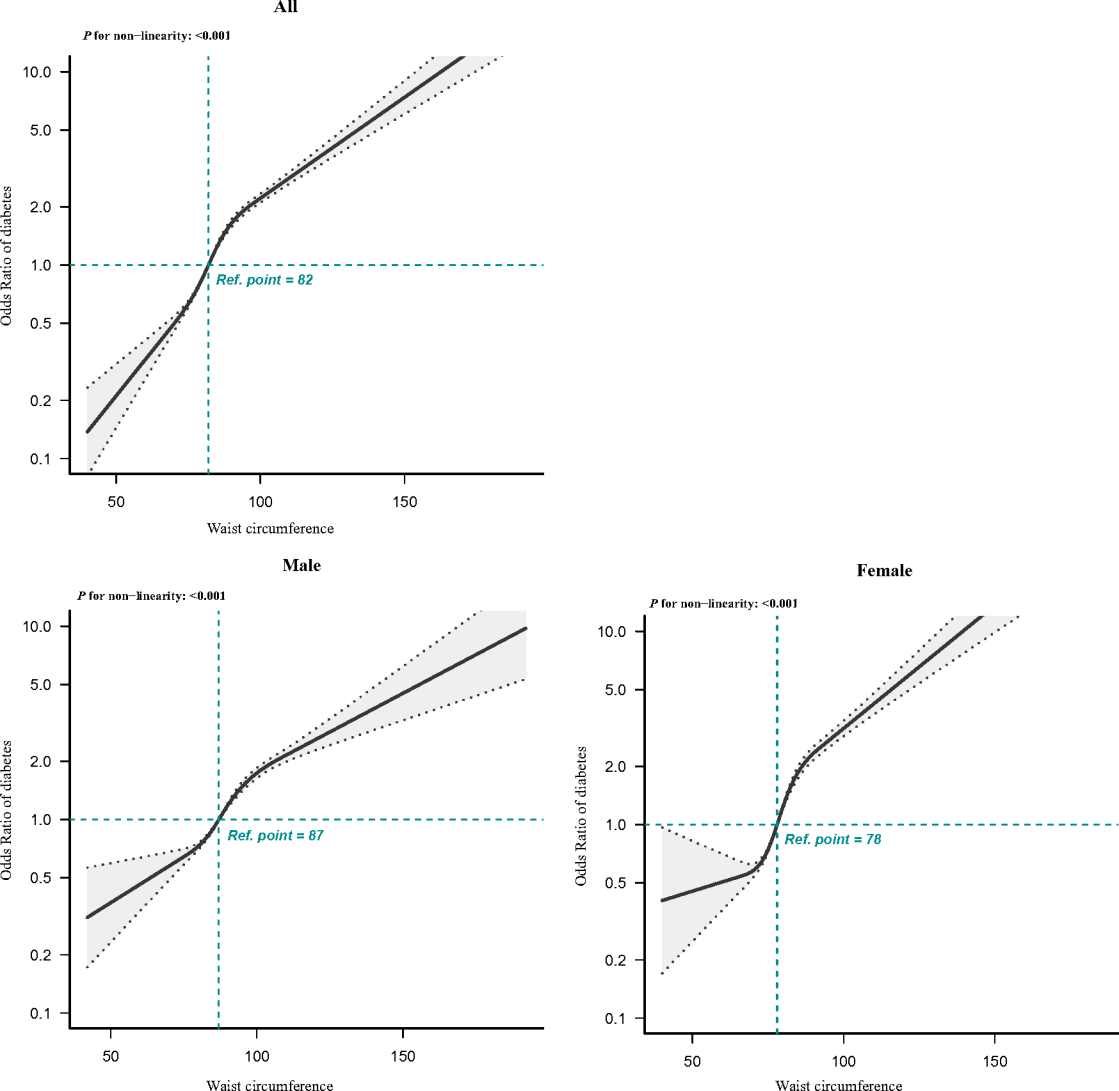
RCS of the association between waist circumference and T2DM. The association was adjusted for gender, age, SBP, DBP, coronary atherosclerotic heart disease, stroke, and family history of diabetes. Gender was not adjusted in the RCS regression analysis stratified by gender. T2DM, type 2 diabetes mellitus; RCS, restricted cubic spline; SBP, systolic blood pressure; DBP, diastolic blood pressure.

In multivariate logistic regression analyses, patients with abdominal obesity presented 2.817 times higher risk of T2DM in comparison with those with non-abdominal obesity (OR, 2.817; 95% CI, 2.700–2.939). After additional adjustment for age, gender, SBP, DBP, coronary atherosclerotic heart disease, stroke, and family history of diabetes, abdominal obesity remained significantly related to T2DM (**Table 3**). The relationship between abdominal obesity and T2DM was consistent in both men and women after adjusting for confounding factors in Model 3. Furthermore, the RCS plot for analyzing the relationship between waist circumference and the risk of T2DM was exhibited in **Figure 2**. The shape of the dose–response correlation between waist circumference and diabetes risk was non-linear (*P* for non-linearity < 0.001). We found that, when waist circumference was about 82 cm, the OR corresponding to the risk of T2DM was about 1; when waist circumference was greater than 82 cm, the risk of T2DM increased gradually. As waist circumference elevated, the positive relationship between waist circumference and T2DM risk became steeper when waist circumference exceeded 78 cm for women (*P* for non-linearity < 0.001).

**Table 3:** Effect of abdominal obesity on T2DM.

		OR	95% CI	*P*-value
All subjects	Crude model	2.817	2.700–2.939	<0.001
	Model 1	2.429	2.326–2.537	<0.001
	Model 2	2.120	2.028–2.216	<0.001
	Model 3	2.062	1.969–2.161	<0.001
Male	Crude model	2.125	2.013–2.243	<0.001
	Model 1	2.106	1.995–2.223	<0.001
	Model 2	1.874	1.773–1.981	<0.001
	Model 3	1.798	1.696–1.905	<0.001
Female	Crude model	3.329	3.106–3.569	<0.001
	Model 1	3.067	2.859–3.291	<0.001
	Model 2	2.606	2.425–2.800	<0.001
	Model 3	2.607	2.418–2.811	<0.001

Model 1: Adjusted for age and gender.

Model 2: Adjusted for age, gender, SBP, and DBP.

Model 3: Adjusted for age, gender, SBP, DBP, coronary atherosclerotic heart disease, stroke, and family history of diabetes.

Gender was not adjusted in the regression analyses stratified by gender. The non-abdominal obesity was defined as the referent category.

T2DM, type 2 diabetes mellitus; SBP, systolic blood pressure; DBP, diastolic blood pressure; OR, odds ratios; CI, confidence interval.

### Regression-based mediation analysis of adiponectin on association of abdominal obesity with T2DM

**Figure 3:**
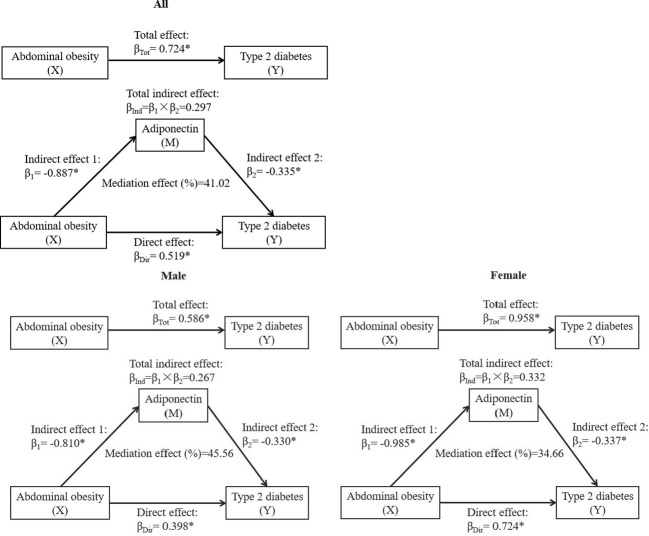
The mediation effect of adiponectin on the association between abdominal obesity and T2DM. β, standardized regression coefficient; β_1_, indirect effect 1; β_2_, indirect effect 2; β_Ind_, total indirect effect; β_Dir_, direct effect; β_Tot_, total effect. X, independent variable; Y, dependent variable; M, mediator. *P < 0.001. Models were adjusted for age, gender, SBP, DBP, coronary atherosclerotic heart disease, stroke, and family history of diabetes. Gender was not adjusted in the mediation effect analysis stratified by sex.

The mediation effect of adiponectin on the association between abdominal obesity and T2DM is shown in **Figure 3**. The total effect of abdominal obesity on T2DM was notable according to a standardized regression coefficient (β_Tot_ = 0.724, *P* < 0.001). The abdominal obesity was negatively correlated with circulating adiponectin (β_1_ = −0.887, *P* < 0.001). The total indirect effect through adiponectin defined as the product of β_1_ and β_2_ was significant (β_Ind_ = 0.297, *P* < 0.001). The mediation effect of adiponectin on abdominal obesity–T2DM association was 41.02% after adjusting the confounders, including age, gender, SBP, DBP, coronary atherosclerotic heart disease, stroke, and family history of diabetes. Similarly, the mediation effects of adiponectin levels on the association between abdominal obesity and T2DM were significant in both genders, with the mediation effect of 45.56% in men and 34.66% in women.

## Discussion

This study demonstrated a non-linear and positive correlation between waist circumference and the risk of diabetes in the Chinese population. In addition, we also found that abdominal obesity was closely related to T2DM through circulating adiponectin. Our study highlighted that the secretory adiponectin played an important intermediary role in obesity-induced T2DM, and this mediation effect accounts for a relatively large proportion of T2DM (41.02%). The level of circulating adiponectin could be used to represent the risk of obesity-induced diabetes.

It has been generally accepted that adiposity is closely related to various obesity-related chronic metabolic diseases, especially T2DM (17). The current opinion recommended that waist circumference threshold for central obesity is a useful indicator for the detection of obesity-related diabetes. Numerous previous studies have revealed that waist circumference can serve as an excellent instrument for detecting T2DM (18, 19). Consistently, we also found that abdominal obesity was significantly correlated with increased odds of T2DM in Chinese population. As an important endocrine organ, adipose tissue is responsible for not only fat storage but also releasing a great number of adipokines that play important roles in the pathogenesis of various metabolic disorders (20). Thus, the relationship between central obesity and T2DM may be attributed to the adipose that regulates metabolic homeostasis by secreting adipocytokines.

Several adipokines secreted from adipocytes, including adiponectin, have been identified to play an essential role in modulating lipid and glucose metabolic homeostasis. Of note, though, adiponectin differs from other secretory adipokines in that it decreases in patients with obesity and it is inversely proportional to the distribution of body fat (21). Consistently, our data also showed that the circulating adiponectin level was negatively associated with BMI and waist circumference. Moreover, previous studies reported that the serum adiponectin was negatively correlated with plasma insulin and might be a primary protective factor in the development of T2DM. Coltell et al. suggested that the level of plasma adiponectin was not only strongly related to HDL-C and triglycerides but also inversely associated with fasting glucose and T2DM (22). Our results also indicated that adiponectin was inversely related to FPG, and patients with T2DM also presented with a lower level of circulating adiponectin (data were not shown). However, it was still unclear to whether the effect of adiposity on T2DM is mediated by circulating adiponectin. Of note, our mediating effect model demonstrated that central obesity had an adverse effect on T2DM, which was partially mediated by the decreased adiponectin, with a mediation effect of 41.02% in the central obesity–T2DM association. This mediating effect may be explained, in part, by the major function of adiponectin, which not only increased fatty acid oxidation and glucose utilization in the skeletal muscle and liver but also increased insulin sensitivity through increasing peroxisome proliferator–activated receptor–α activity and AMP-activated protein kinase (23). The present study suggested that the expansion of abdominal adipose tissue decreased the circulating adiponectin, which may have a potential impact on the occurrence and development of T2DM. However, the exact mechanisms of adiponectin in the pathogenesis of obesity-induced T2DM remain for further investigation *in vitro* and *in vivo*. Nonetheless, this observation provided additional and strong evidence that the fat-secreted factor, adiponectin, is involved in cross talk between abdominal obesity and obesity-related diabetes. In the future clinical and epidemiological practices, patients with obesity with low circulating adiponectin levels can be recognized as a high-risk group for T2DM.

The novelty of this large-scale, population-based study is that we evaluated and further quantified the mediating effects of circulating adiponectin on the association between abdominal obesity and T2DM. We found that the path from central obesity and T2DM was adversely mediated by adiponectin, suggesting that elevating adiponectin secretion may be the therapeutic potentials for protecting obesity from T2DM. These findings demonstrated that adiponectin could be served as a monitoring marker for weight management or lifestyle intervention in the future. However, some limitations also existed in the present study. Firstly, because of the cross-sectional study design, we are unable to exclude the existence of inverse direction in the pathways of association. Thus, the pathway of the association needs to be further corroborated in future prospective studies with long follow-up periods. Secondly, we did not distinguish the type of diabetes in surveys. However, because T2DM accounts for more than 90% of all diabetes diagnosed cases (24), our finding is likely more representative of T2DM. Thirdly, although we adjusted for a series of confounders, residual confounding due to the measurement errors in evaluation and unmeasured factors such as dietary and physical activity cannot be excluded. Moreover, we did not further explore the potential role of other markers, such as leptin, insulin resistance, lipids, and visceral adiposity in this mediating effect analysis. Thus, the interaction between these factors needs to be further investigated in future studies. Finally, our analysis was performed on the Chinese adults who lived in Dongguan, Guangdong Province, which limits the generalizability to other geographic regions or ethnicities; however, given its large sample size and wide age range, the results of our present study will have wide applicability for the population in China.

## Conclusion

In this study, abdominal obesity was significantly related to T2DM through decreased circulating adiponectin level, which was strong and applied to both men and women. The quantification of the mediation effect supports an intermediary role of adiponectin in the occurrence and development of T2DM among population with abdominal obesity. Thus, our finding may help to facilitate development of the novel prevention and therapeutic strategies for controlling the adverse progression from obesity to T2DM in future practice.

## Data availability statement

The raw data supporting the conclusions of this article will be made available by the authors, without undue reservation.

## Ethics statement

The studies involving humans were approved by the tenth Affiliated Hospital of Southern Medical University (Dongguan People’s Hospital). The studies were conducted in accordance with the local legislation and institutional requirements. The participants provided their written informed consent to participate in this study.

## Author contributions

LH: Conceptualization, Data curation, Formal analysis, Investigation, Methodology, Project administration, Resources, Software, Validation, Visualization, Writing – original draft, Writing – review & editing. WX: Conceptualization, Data curation, Formal analysis, Investigation, Methodology, Project administration, Resources, Software, Validation, Visualization, Writing – original draft, Writing – review & editing. DL: Data curation, Formal analysis, Investigation, Methodology, Software, Validation, Visualization, Writing – original draft, Writing – review & editing. JZ: Data curation, Formal analysis, Investigation, Methodology, Validation, Writing – original draft, Writing – review & editing. HL: Formal analysis, Investigation, Methodology, Validation, Writing – review & editing. HC: Investigation, Methodology, Supervision, Validation, Writing – review & editing. XZ: Conceptualization, Funding acquisition, Project administration, Supervision, Writing – review & editing. WC: Conceptualization, Data curation, Formal analysis, Investigation, Methodology, Project administration, Resources, Supervision, Validation, Writing – original draft, Writing – review & editing.

## Supplementary material

Supplementary Figure 1 Mediation analysis model. β, standardized regression coefficient; β_1_, indirect effect 1; β_2_, indirect effect 2; β_Ind_, total indirect effect; β_Dir_, direct effect; β_Tot_, total effect.
