# Supplementary figures and images for: The role of adiponectin in the association between abdominal obesity and type 2 diabetes: a mediation analysis among 232,438 Chinese participants
Source: Front Endocrinol (Lausanne). 2024 Feb 22;15:1327716. doi: 10.3389/fendo.2024.1327716 (PMC10919146; doi:10.3389/fendo.2024.1327716)

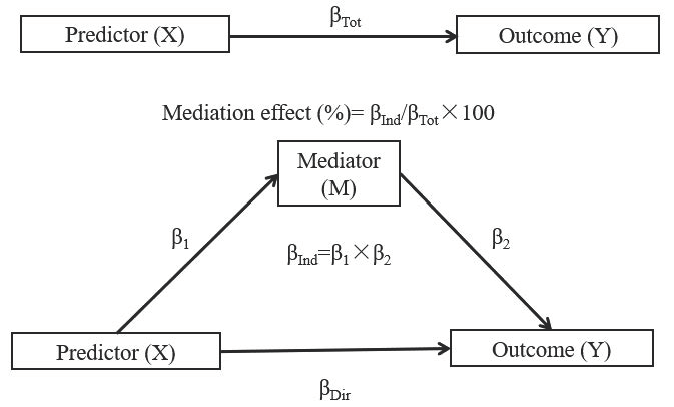

Supplement: Supplementary Figure 1 — Mediation analysis model. β, standardized regression coefficient; β1, indirect effect 1; β2, indirect effect 2; βInd, total indirect effect; βDir, direct effect; βTot, total effect. [file Image_1.tif]
